# The Diagnostic Ability of GPT-3.5 and GPT-4.0 in Surgery: Comparative Analysis

**DOI:** 10.2196/54985

**Published:** 2024-09-10

**Authors:** Jiayu Liu, Xiuting Liang, Dandong Fang, Jiqi Zheng, Chengliang Yin, Hui Xie, Yanteng Li, Xiaochun Sun, Yue Tong, Hebin Che, Ping Hu, Fan Yang, Bingxian Wang, Yuanyuan Chen, Gang Cheng, Jianning Zhang

**Affiliations:** 1 Department of Neurosurgery The First Medical Centre Chinese PLA General Hospital Beijing China; 2 Department of Respiratory and Critical Care Medicine The First Medical Centre Chinese PLA General Hospital Beijing China; 3 Department of Neurosurgery Sanmenxia Central Hospital Sanmenxia China; 4 School of Health Humanities Peking University Beijing China; 5 Medical Innovation Research Division Chinese People's Liberation Army General Hospital Beijing China; 6 National Engineering Research Center for Medical Big Data Application Technology Chinese People's Liberation Army General Hospital Beijing China; 7 Departments of Urology The First Affiliated Hospital of Fujian Medical University Fuzhou China

**Keywords:** ChatGPT, accuracy rates, artificial intelligence, diagnosis, surgeon

## Abstract

**Background:**

ChatGPT (OpenAI) has shown great potential in clinical diagnosis and could become an excellent auxiliary tool in clinical practice. This study investigates and evaluates ChatGPT in diagnostic capabilities by comparing the performance of GPT-3.5 and GPT-4.0 across model iterations.

**Objective:**

This study aims to evaluate the precise diagnostic ability of GPT-3.5 and GPT-4.0 for colon cancer and its potential as an auxiliary diagnostic tool for surgeons and compare the diagnostic accuracy rates between GTP-3.5 and GPT-4.0. We precisely assess the accuracy of primary and secondary diagnoses and analyze the causes of misdiagnoses in GPT-3.5 and GPT-4.0 according to 7 categories: patient histories, symptoms, physical signs, laboratory examinations, imaging examinations, pathological examinations, and intraoperative findings.

**Methods:**

We retrieved 316 case reports for intestinal cancer from the Chinese Medical Association Publishing House database, of which 286 cases were deemed valid after data cleansing. The cases were translated from Mandarin to English and then input into GPT-3.5 and GPT-4.0 using a simple, direct prompt to elicit primary and secondary diagnoses. We conducted a comparative study to evaluate the diagnostic accuracy of GPT-4.0 and GPT-3.5. Three senior surgeons from the General Surgery Department, specializing in Colorectal Surgery, assessed the diagnostic information at the Chinese PLA (People’s Liberation Army) General Hospital. The accuracy of primary and secondary diagnoses was scored based on predefined criteria. Additionally, we analyzed and compared the causes of misdiagnoses in both models according to 7 categories: patient histories, symptoms, physical signs, laboratory examinations, imaging examinations, pathological examinations, and intraoperative findings.

**Results:**

Out of 286 cases, GPT-4.0 and GPT-3.5 both demonstrated high diagnostic accuracy for primary diagnoses, but the accuracy rates of GPT-4.0 were significantly higher than GPT-3.5 (mean 0.972, SD 0.137 vs mean 0.855, SD 0.335; *t*_285_=5.753; *P*<.001). For secondary diagnoses, the accuracy rates of GPT-4.0 were also significantly higher than GPT-3.5 (mean 0.908, SD 0.159 vs mean 0.617, SD 0.349; *t*_285_=–7.727; *P*<.001). GPT-3.5 showed limitations in processing patient history, symptom presentation, laboratory tests, and imaging data. While GPT-4.0 improved upon GPT-3.5, it still has limitations in identifying symptoms and laboratory test data. For both primary and secondary diagnoses, there was no significant difference in accuracy related to age, gender, or system group between GPT-4.0 and GPT-3.5.

**Conclusions:**

This study demonstrates that ChatGPT, particularly GPT-4.0, possesses significant diagnostic potential, with GPT-4.0 exhibiting higher accuracy than GPT-3.5. However, GPT-4.0 still has limitations, particularly in recognizing patient symptoms and laboratory data, indicating a need for more research in real-world clinical settings to enhance its diagnostic capabilities.

## Introduction

Artificial intelligence (AI) can potentially improve health care outcomes as a tool in various fields of medicine [1]. While its role in medicine has thus far been limited to specific cases, it has not been widely implemented in clinical practice. Recently, OpenAI released an AI-powered ChatGPT for public use [2]. ChatGPT is a powerful neural network model belonging to the GPT family of large language models (LLMs). Despite being created primarily for humanlike conversations, ChatGPT has shown remarkable versatility and has the potential to revolutionize many industries [3]. Its release has garnered widespread attention for potential use in numerous fields, especially in medicine [4-6].

Recently, GPT-4.0, an upgraded version of GPT-3.5, demonstrated remarkable improvements in professional and academic benchmarks, including the US Medical Licensing Examination [7]. We previously used ChatGPT to answer medical questions and compared ChatGPT’s abilities with evidence-based neurosurgeons [8]. Unlike low-seniority surgeons, ChatGPT has no lack of foundational knowledge and is not limited by cognitive load, especially GPT-4.0, which was comparable to that of surgeons with high seniority [8]. It also showed that ChatGPT generates semantically accurate responses to text-based questions, including medical textual data, making it a highly potential candidate for clinical decision support (CDS) applications.

Colorectal cancer is a type of cancer that affects the colon (large intestine) or rectum [9], which is the third most common cancer and the second leading cause of cancer-related deaths worldwide, accounting for approximately 10% of all cancer cases [9]. Colon cancer is a kind of disease with very obvious symptoms including diarrhea, constipation, blood in the stool, abdominal pain, unexplained weight loss, fatigue, and low iron levels [10]. A diagnosis can be made once a surgeon recognizes that a patient has these symptoms.

Surgeons usually use clinical experience to make diagnoses based on patient’s symptoms, examination results, medical histories, physical signs, and intraoperative findings. This process will consume much time to occupy the workforce and will be subjective. Misdiagnoses or delays in diagnosis may occur. Innovative approaches to improve diagnostic accuracy and efficiency are needed. Many AI models have been used in health care to aid CDS [11-13]. However, few studies still exist on how ChatGPT can read and analyze clinical case text data and provide diagnoses based on real-world clinical information obtained at the hospital.

This study investigates the use of ChatGPT for diagnosis and assesses its performance. Furthermore, substantial performance improvement indicates that LLMs are quickly approaching readiness for use in the clinical setting. Continuous evaluation is necessary to keep pace with model progress. Therefore, we also compared the performance of two versions of ChatGPT (GPT-4.0 and GPT-3.5) to understand the impact of model updates on performance.

## Methods

### Study Design

We designed a comparative study to evaluate the applicability of ChatGPT (GPT-4.0 and GPT-3.5) in surgery using clinical information from the case database of the Chinese Medical Association Publishing House (CMAPH). GPT-3.5 is a free, widely accessible platform, while GPT-4.0 is a newer release claiming better performance, allowing us to evaluate improvements in GPT-4.0 comprehensively. The CMAPH database is a comprehensive and authoritative resource. It provides access to current and archived issues across various medical specialties, making it invaluable for researchers, clinicians, and health care professionals [14]. This study used colorectal cancer–related literature from this database, leveraging its extensive repository to support our analysis.

### Study Data

A total of 316 case reports for intestinal cancer between August 1982 and June 2023 were retrieved from the CMAPH database by a researcher (CY); then, FY extracted the case records from the literature and designed a standardized data organization form by Excel (Microsoft Corp), which included extracted case records, three response records from GPT-3.5 and GPT-4.0, an evaluation section for the responses from GPT-3.5 and GPT-4.0, and a detailed assessment of seven categories of misdiagnoses. Another researcher (BW) then conducted a thorough data cleansing process, removing cases with incomplete information, excluding diagnosis-related data from the literature, and translating the data according to clinical terminology. JL was responsible for verifying the translation results. We applied the World Health Organization’s *ICD-11* (*International Classification of Diseases, 11th Revision*) diagnostic criteria [15,16] to these cases, excluding cases where the diagnosis and related information conflicted with the *ICD-11* standards. This process resulted in a final count of 286 valid cases for analysis. The collected data included age, gender, medical histories, symptoms, physical signs, laboratory examinations, imaging examinations, pathological examinations, and intraoperative findings, all structured as text data. Patient identities were kept confidential and anonymized for the analysis.

### Prompt Design and Input Strategies

We aimed for GPT-3.5 and GPT-4.0 to provide appropriate primary and secondary diagnoses based on the case information from the literature data. Therefore, we designed the prompt in English, “Please provide the most likely primary and secondary diagnoses,” and conducted tests accordingly. We randomly selected 5 of the 286 cases for initial testing, using the designed prompt to query GPT-3.5 and GPT-4.0 thrice each. It was a success if both models provided diagnoses without refusing to answer. JZ conducted these tests, and the answers obtained were recorded in the standardized data form. Subsequently, these 5 cases were not queried again. During these tests, both GPT-3.5 and GPT-4.0 successfully provided answers as required. Consequently, we finalized this prompt for use in this study. A researcher (JZ) used the same computer device to query GPT-3.5 and GPT-4.0 from August 1, 2023, to November 1, 2023, while another researcher (YT) was responsible for proofreading and verification.

Due to the potential for inconsistent responses from ChatGPT when the same question is asked multiple times, we posed the prompt for each case three times to GPT-3.5 and GPT-4.0. In this study, the prompt for each case was first posed three times using GPT-3.5, followed by three times using GPT-4.0, without providing any feedback to either model after each inquiry. To avoid any contextual learning that could affect the accuracy of the responses, each inquiry to GPT-3.5 and GPT-4.0 was conducted in a new chat session. The mean accuracy rate represents the average accuracy derived from these three inquiries for each model. We conducted the Fleiss κ test on the primary and secondary diagnostic responses provided by GPT-3.5 and GPT-4.0, defining consistency as the ability to provide the same information with each submission.

### Data Measurement

The accuracy of GPT-3.5 and GPT-4.0 in providing primary and secondary diagnoses was evaluated based on explicit diagnoses in published literature. Diagnoses that matched the explicit diagnoses in the literature were recorded as “correct,” while those that did not were recorded as “incorrect.” An accuracy rate exceeding 0.800 for GPT-4.0 was deemed acceptable, while an accuracy rate exceeding 0.600 for GPT-3.5 was considered acceptable [17-19].

We assessed the diagnostic errors made by GPT-3.5 and GPT-4.0, categorizing them into 7 types: medical histories, symptoms, physical signs, laboratory examinations, imaging examinations, pathological examinations, and intraoperative findings. Due to the literature case records containing these 7 aspects, we discussed and decided to categorize the diagnostic errors accordingly. The criteria for each category are as follows: (1) medical histories: past medical conditions, treatments, surgeries, and other relevant health information; (2) symptoms: subjective indications reported by the patient, such as pain, fever, changes in bowel habits, rectal bleeding, abdominal pain, unexpected weight loss, and fatigue; (3) physical signs: objective observations during a physical examination including abdominal tenderness, palpable masses, anemia, or ascites; (4) laboratory examination: analysis of biological samples including blood chemistry, hematology, urinalysis, complete blood count for anemia, stool tests for occult blood, and tumor markers like carcinoembryonic antigen; (5) imaging examination: techniques to create internal body images such as x-rays, computed tomography scans, magnetic resonance imaging, ultrasound, and barium enemas; (6) pathological examination: microscopic analysis of tissue samples to determine cancer type and differentiation; and (7) intraoperative findings: observations during surgery regarding tumor location, size, extent, and involvement of adjacent organs or lymph nodes. For instance, if a diagnosis is primarily based on the patient’s medical history and ChatGPT’s response is incorrect, the error pattern is classified as a history-based error. If the diagnosis derives from multiple sources and ChatGPT provides an incorrect answer, we categorize all relevant sources as contributing to the error pattern.

We selected 3 senior surgeons not involved in this study to independently evaluate the diagnostic information and determine the primary and secondary diagnoses. All three senior surgeons are from the General Surgery Department, specializing in Colorectal Surgery, at the Chinese PLA (People’s Liberation Army) General Hospital, each with at least 20 years of clinical experience. The National Health Commission of the People’s Republic of China defines senior surgeons as chief physicians with over 3 years of experience as associate chief physicians in surgery. The primary and secondary diagnoses provided by GPT-3.5 and GPT-4.0 were scored based on the previously mentioned accuracy assessment criteria. For the primary diagnosis, responses were scored as “correct” with a value of 1 and “incorrect” with a value of 0. For secondary diagnoses, the scoring was based on the ratio of the number of correct secondary diagnoses provided by ChatGPT to the total number of correct secondary diagnoses in the literature. For example, if there were five correct secondary diagnoses and ChatGPT provided three correct ones, the score would be 3/5. The diagnostic errors of GPT-3.5 and GPT-4.0 were categorized according to the error mentioned above pattern classification criteria. Errors involving a category were assigned a value of 1, while noninvolvement was assigned a value of 0. The three senior surgeons used the standardized data recording form to document their evaluations, and discrepancies were discussed in meetings held at the hospital to reach a consensus on the scoring methodology to ensure consistency and accuracy in the evaluation process, with interrater agreement assessed using Cohen κ statistic.

### Statistical Analysis

The data were normally distributed as determined by the Kolmogorov-Smirnov test. We analyzed data using SPSS (version 25.0; IBM Corp). We used mean (SD) values to represent numerical variables and describe qualitative variables using absolute values of special group cases. The statistical methods are mainly based on *t* tests, and repeated measures analysis of variance is used for statistical analysis. The difference is statistically significant with *P*<.05.

### Ethical Considerations

No ethical approval or informed consent was required for this study, as it used publicly available data. All study designs strictly adhere to the Guidelines and Checklist for the Reporting on Digital Health Implementations checklist [20]. This study does not use AI to generate any related content.

## Results

### Baseline Characteristics

**Figure 1:**
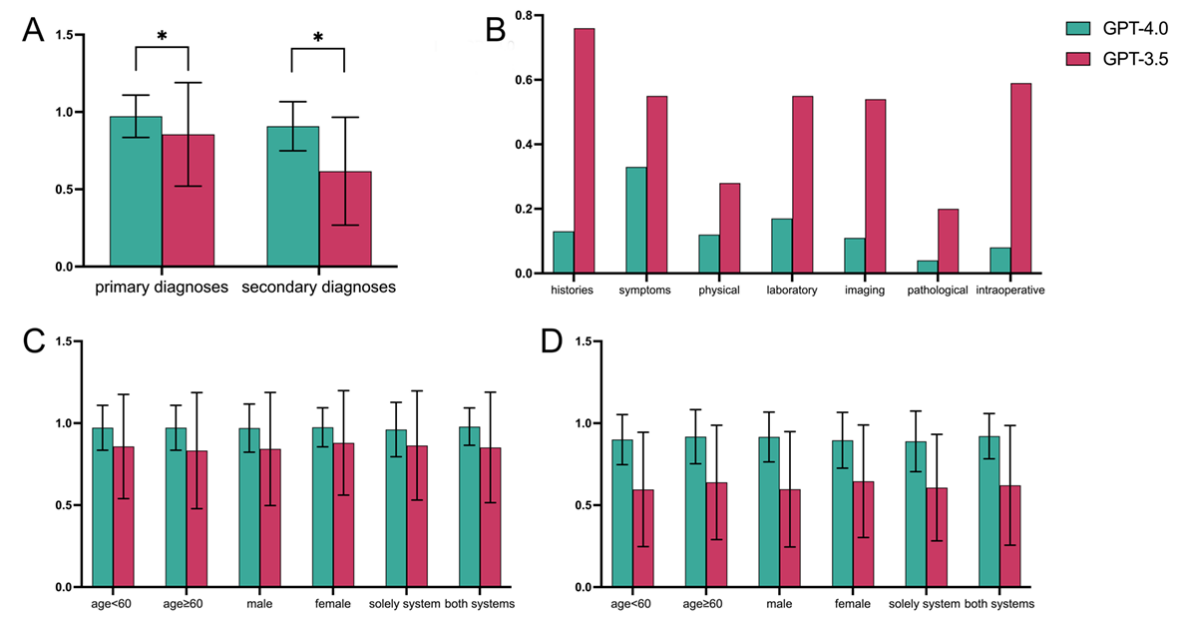
The comparison of GPT-4.0 and GPT-3.5. (A) The comparison of GPT-4.0 and GPT-3.5 in diagnostic accuracy for the primary and secondary diagnoses. (B) The comparison of GPT-4.0 and GPT-3.5 in error patterns (medical histories, symptoms, physical signs, examination results, and intraoperative findings). (C) The comparison of GPT-4.0 and GPT-3.5 in age, gender, and system group for the primary diagnoses. (D) The comparison of GPT-4.0 and GPT-3.5 in age, gender, and system group for the secondary diagnoses.

Detailed questions and responses are included in Multimedia Appendix 1 (Figure S1 in Multimedia Appendix 1). Cohen κ ranged between moderate to near perfect agreement (Multimedia Appendix 2). For primary diagnoses, the mean accuracy rates of GPT-4.0 were 0.972 (SD 0.137), and GPT-3.5 were 0.855 (SD 0.335). The accuracy rates of GPT-4.0 were significantly higher than GPT-3.5 (*t*_285_=5.753; *P*<.001). For secondary diagnoses, the mean accuracy rate of GPT-4.0 was 0.908 (SD 0.159), and GPT-3.5 was 0.617 (SD 0.349). The accuracy rates of GPT-4.0 were also significantly higher than GPT-3.5 (*t*_285_=–7.727; *P*<.001; Table 1 and Figure 1A).

We solved any discrepancies. Of the 286 cases, 19 cases exhibited inconsistencies, while 267 cases were consistent, resulting in an agreement rate of 93.36%. The discrepancy rate stands at 6.64%. The three senior surgeons demonstrated excellent agreement in their assessments (Multimedia Appendix 3). We analyzed the ChatGPT performance according to error patterns. The rates of diagnosis errors due to medical histories in GPT-4.0 were significantly lower than in GPT-3.5 (mean 0.13, SD 0.520 vs mean 0.76, SD 1.446; *t*_285_=–3.384; *P*<.001). The rates of diagnosis errors due to symptoms in GPT-4.0 were significantly lower than in GPT-3.5 (mean 0.33, SD 0.877 vs mean 0.55, SD 1.125; *t*_285_=–3.840; *P*=.001). The rates of diagnosis errors due to physical signs in GPT-4.0 were significantly lower than in GPT-3.5 (mean 0.12, SD 0.516 vs mean 0.28, SD 0.867; *t*_285_=–5.959; *P*<.001). The rates of laboratory examination due to medical histories in GPT-4.0 were significantly lower than in GPT-3.5 (mean 0.17, SD 0.645 vs mean 0.55, SD 1.131; *t*_285_=–6.738; *P*<.001). The rates of diagnosis errors due to imaging examination in GPT-4.0 were significantly lower than in GPT-3.5 (mean 0.11, SD 0.483 vs mean 0.54, SD 1.125; *t*_285_=–6.846; *P*<.001). The rates of diagnosis errors due to pathological examination in GPT-4.0 were significantly lower than in GPT-3.5 (mean 0.04, SD 0.294 vs mean 0.20, SD 0.739; *t*_285_=–3.536; *P*<.001). The rates of diagnosis errors due to imaging examination in GPT-4.0 were significantly lower than in GPT-3.5 (mean 0.08, SD 0.403 vs mean 0.59, SD 1.258; *t*_285_=14.006; *P*<.001; Table 1 and Figure 1B).

**Table 1:** Baseline characteristics.

Analyzed pairs			GTP-4.0, mean (SD)		GTP-3.5, mean (SD)		*t* test (*df*)		*P* value
Primary diagnoses			0.972 (0.137)		0.855 (0.335)		5.753 (285)		<.001
Secondary diagnoses			0.908 (0.159)		0.617 (0.349)		–7.727 (285)		<.001
**Error patterns**									
	Medical histories	0.13 (0.520)		0.76 (1.446)		–3.384 (285)		<.001	
	Symptoms	0.33 (0.877)		0.55 (1.125)		–3.840 (285)		.001	
	Physical signs	0.12 (0.516)		0.28 (0.867)		–5.959 (285)		<.001	
	Laboratory examination	0.17 (0.645)		0.55 (1.131)		–6.738 (285)		<.001	
	Imaging examination	0.11 (0.483)		0.54 (1.125)		–6.846 (285)		<.001	
	Pathological examination	0.04 (0.294)		0.20 (0.739)		–3.536 (285)		<.001	
	Intraoperative findings	0.08 (0.403)		0.59 (1.258)		14.006 (285)		<.001	

### Comparison of Accuracy Rates According to Specialty Classification in Primary Diagnoses

Regarding age groups, there was no significant difference between ages of <60 years and ≥60 years in GPT-4.0 (mean 0.972, SD 0.137 vs mean 0.972, SD 0.137; *t*_284_=0.002; *P*=.99), and also no significant difference between them in GPT-3.5 (mean 0.875, SD 0.318 vs mean 0.832, SD 0.354; *t*_284_=1.086, *P*=.28). In the gender group, there was no significant difference between male and female in GPT-4.0 (mean 0.970, SD 0.147 vs mean 0.975, SD 0.119; *t*_284_=–0.291; *P*=.77), and also no significant difference between them in GPT-3.5 (mean 0.842, SD 0.345 vs mean 0.879, SD 0.319; *t*_284_=–0.898; *P*=.37). In the diagnostic type group, there was no significant difference between “solely intradigestive system” and “both intradigestive and extradigestive system” in GPT-4.0 (mean 0.961, SD 0.166 vs mean 0.979, SD 0.114; *t*_284_=–1.063; *P*=.29), and also no significant difference between them in GPT-3.5 (mean 0.863, SD 0.333 vs mean 0.851, SD 0.337; *t*_284_=0.308; *P*=.76; Table 2). Moreover, there was a significant difference between GPT-4.0 and GPT-3.5 in age, gender, and diagnostic type group (Figure 1C).

**Table 2:** Comparison of accuracy rates according to specialty classification in primary diagnoses.

Analyzed pairs		GPT-4.0, mean (SD)	GPT-3.5, mean (SD)	*t* test (*df*)	*P* value			
**Age (in years)^a^**								
	<60	0.972 (0.137)	0.875 (0.318)	3.688 (154)	<.001			
	≥60	0.972 (0.137)	0.832 (0.354)	4.445 (130)	<.001			
**Sex^b^**								
	Male	0.970 (0.147)	0.842 (0.345)	3.025 (106)	.003			
	Female	0.975 (0.119)	0.879 (0.319)	4.910 (178)	<.001			
**Diagnostic type^c^**								
	Solely intradigestive system	0.961 (0.166)	0.863 (0.333)	2.983 (111)	.004			
	Both intradigestive and extradigestive system	0.979 (0.114)	0.851 (0.337)	4.991 (173)	<.001			

^a^In the age group, GPT-4.0 *t*_284_=0.002 and *P*=.99; GPT-3.5 *t*_284_=1.086 and *P*=.28.

^b^In the gender group, GPT-4.0 *t*_284_=–0.291 and *P*=.77; GPT-3.5 *t*_284_=–0.898 and *P*=.37.

^c^In the diagnostic type group, GPT-4.0 *t*_284_=–1.063 and *P*=.29; GPT-3.5 *t*_284_=0.308 and *P*=.76.

### Comparison of Accuracy Rates According to Specialty Classification in Secondary Diagnoses

Regarding age group, there was no significant difference between ages of <60 years and ≥60 years in GPT-4.0 (mean 0.900, SD 0.153 vs mean 0.918, SD 0.165; *t*_263_=–0.910; *P*=.36), and also no significant difference between them in GPT-3.5 (mean 0.596, SD 0.349 vs mean 0.639, SD 0.349; *t*_264_=–1.006; *P*=.32). In the gender group, there was no significant difference between male and female in GPT-4.0 (mean 0.916, SD 0.152 vs mean 0.896, SD 0.170; *t*_263_=0.972; *P*=.33), and also no significant difference between them in GPT-3.5 (mean 0.597, SD 0.352 vs mean 0.646, SD 0.343; *t*_264_=–1.114; *P*=.27). In the diagnostic type group, there was no significant difference between “solely intradigestive system” and “both intradigestive and extradigestive system” in GPT-4.0 (mean 0.889, SD 0.185 vs mean 0.921, SD 0.138; *t*_263_=–1.569; *P*=.12), and also no significant difference between them in GPT-3.5 (mean 0.607, SD 0.325 vs mean 0.621, SD 0.365; *t*_264_=–0.333; *P*=.74; Table 3). Moreover, there was a significant difference between GPT-4.0 and GPT-3.5 in age, gender, and diagnostic type groups (Table 3 and Figure 1D).

**Table 3:** Comparison of accuracy rates according to specialty classification in secondary diagnoses.

Analyzed pairs		GPT-4.0, mean (SD)	GPT-3.5, mean (SD)	*t* test *(df*)	*P* value	
**Age (in years)^a^**						
	<60	0.900 (0.153)	0.596 (0.349)	10.764 (142)	<.001	
	≥60	0.918 (0.165)	0.639 (0.349)	8.974 (121)	<.001	
**Sex^b^**						
	Male	0.916 (0.152)	0.597 (0.352)	7.902 (99)	<.001	
	Female	0.896 (0.170)	0.646 (0.343)	11.613 (164)	<.001	
**Diagnostic type^c^**						
	Solely intradigestive system	0.889 (0.185)	0.607 (0.325)	8.850 (104)	<.001	
	Both intradigestive and extradigestive system	0.921 (0.138)	0.621 (0.365)	10.844 (159)	<.001	

^a^In the age group, GPT-4.0 *t*_263_=–0.910 and *P*=.36; GPT-3.5 *t*_264_=–1.006 and *P*=.32.

^b^In the gender group, GPT-4.0 *t*_263_=0.972 and *P*=.33; GPT-3.5 *t*_264_=–1.114 and *P*=.27.

^c^In the diagnostic type group, GPT-4.0 *t*_263_=–1.569 and *P*=.12; GPT-3.5 *t*_264_=–0.333 and *P*=.74.

## Discussion

### Principal Findings

This comparative study evaluated the diagnostic capabilities of ChatGPT, specifically comparing the performance of GPT-4.0 and GPT-3.5 using clinical information from a public case database. Our analysis focused on specialty classification and error patterns including medical histories, symptoms, physical signs, examination results, and intraoperative findings. The results indicated that GPT-4.0 achieved significantly higher accuracy rates for primary and secondary diagnoses than GPT-3.5. Despite improvements, both versions exhibited limitations in processing patient history, symptom presentation, and laboratory test data, with GPT-4.0 showing better performance overall. These findings are consistent with the performance evaluations of GPT-3.5 and GPT-4.0 by Taloni et al [21], Yang et al [22], Deng et al [23], and Antaki et al [24]. However, challenges persist in accurately identifying symptoms and interpreting laboratory results. These findings underscore ChatGPT’s potential in medical diagnosis, highlighting the need for further research to enhance its performance in dynamic clinical environments. This study contributes to ongoing efforts to optimize ChatGPT as an auxiliary tool in clinical practice, emphasizing the importance of continual refinement to ensure its efficacy and reliability in supporting health care professionals’ diagnostic decision-making.

ChatGPT’s ability to read and analyze is helpful in multiple clinical disciplines. Howard et al [25] asked ChatGPT for antimicrobial advice and pointed out that despite no specific clinical advice training, ChatGPT provides compelling responses to most prompts. Nastasi et al [26] found that ChatGPT is currently useful for providing background knowledge on general clinical topics in an emergency. Training ChatGPT with medical corpora, incorporating clinician-supervised feedback, and enhancing its awareness of uncertainty and information-seeking behaviors may improve the medical advice provided by LLMs. Lukac et al [27] evaluated ChatGPT as an adjunct for decision-making in primary breast cancer cases and stated that the eloquence of ChatGPT based on several scientific databases could result in more precise suggestions. Eggmann and Blatz [28] analyzed the chances and challenges of using ChatGPT in dentistry, they found that the use of ChatGPT and similar LLMs in dentistry could streamline administrative workflows and potentially serve as an additional tool for CDS in the future. Kuroiwa et al [29] discussed that the integration of AI, natural language processing, and GPT technologies holds immense promise in the field of psychiatry, which has the potential to revolutionize the way psychiatric disorders are diagnosed, treated, and monitored. Due to the evident symptoms of colon cancer, such as diarrhea, constipation, and blood in the stool, ChatGPT can provide basic, accurate diagnoses based on textual medical records. Previous studies have demonstrated ChatGPT’s strong ability to read and analyze text data. Therefore, in diagnosing colon cancer, ChatGPT can assist in improving doctors’ work efficiency and reducing the workload of initial diagnoses.

Previous studies have evaluated the accuracy of ChatGPT from the perspective of patient self-diagnosis [29], but this study evaluated its accuracy based on clearly defined case record text data from publicly available literature and found its potential to assist in diagnosis, thereby reducing the time required to achieve accurate diagnosis and improving clinical efficiency. The application of ChatGPT in medical diagnosis offers several noteworthy advantages. We set a priori accuracy rate exceeding 0.800 for GPT-4.0 while a priori accuracy rate exceeding 0.600 for GPT-3.5, according to the previous studies [17-19]. In this study, we found that GPT-4.0 has a diagnostic accuracy above 0.900, while GPT-3.5 exhibits an accuracy of over 0.800 in the diagnosis of colon cancer. These results also confirmed that the diagnostic accuracy rates of GPT-3.5 and GPT-4.0 reached acceptable a priori accuracy rates, aligning with previous research findings.

For the primary and secondary diagnoses, the accuracy rates of GPT-4.0 were significantly higher than GPT-3.5. These results suggest significant enhancements in the algorithms and data processing capabilities of GPT-4.0. GPT-4.0 has more advanced neural network architectures, better natural language understanding, and a more extensive and diverse training data set than GPT-3.5, which enables it to handle complex medical diagnostic scenarios effectively [30-33]. GPT-4.0 better contextualizes information and the enhanced ability to understand and process complex medical terminology queries is crucial in clinical diagnosis [23,34]. GPT-4.0’s enhanced ability to contextualize information and process complex medical terminology queries has been instrumental in clinical diagnosis, as it provides a deeper understanding of medical context, terminology, symptom presentation, and laboratory data interpretation [29]. Based on these considerations, the GPT-3.5 to GPT-4.0 upgrade appears effective.

In comparing the classification accuracy of the primary diagnosis, we found that GPT4.0 can achieve high accuracy in disease diagnosis across different ages, genders, and diagnostic type groups. The ability of GPT-4.0 to accurately diagnose patients of all ages and genders is crucial. We believe GPT-4.0 can catch the nuance between different age and gender groups, which can exhibit varying symptoms for the same disease. Besides, previous studies have also presented that GPT-4.0 shows its potential and the richness of its use scenarios in diagnosing a range of diseases across different systems. Hasani et al [35] evaluated the performance of GPT-4.0 in standardizing radiology reports, they indicated that GPT-4.0 could be a reliable tool for generating standardized radiology reports, offering potential benefits such as improved efficiency, better communication, and simplified data extraction and analysis. Liu et al [8] compared the abilities of GPT-4.0 and neurosurgeons, which showed that GPT-4.0’s ability was comparable to that of neurosurgeons with high seniority, and Kanjee et al [36] assessed the diagnostic accuracy of the GPT-4.0 in a series of challenging cases and found that generative AI is a promising adjunct to human cognition in diagnosis. This broad range of diagnostic capabilities could be valuable in clinical practice to assist physicians.

In this study, we categorized the diagnostic errors of GPT-3.5 and GPT-4.0 based on seven aspects covered in the literature: medical histories, symptoms, physical signs, laboratory examinations, imaging examinations, pathological examinations, and intraoperative findings. These 7 aspects are crucial for making definitive diagnoses in clinical practice. Our multidimensional evaluation, therefore, reflects a realistic simulation of clinical application. This approach provides significant clinical value by highlighting the model’s performance across all essential diagnostic dimensions. Such comprehensive assessment ensures that the evaluation of ChatGPT’s diagnostic capabilities is both thorough and relevant to real-world clinical settings. GPT-3.5 has limitations in assessing patient history, symptom presentation, laboratory tests, and imaging data. GPT-3.5 is trained on large data sets, which may not extensively cover specialized medical information, exceptionally detailed patient histories, intricate symptomatology, and complex laboratory or imaging data. GPT-3.5 cannot interpret symptoms, lab results, and imaging data with a nuanced understanding of the patient’s overall clinical picture including history, physical examination, and evolving clinical scenarios as physicians [37]. Furthermore, the knowledge of GPT-3.5 is static. It does not learn in real-time or adapt based on new patient information or the latest medical research, unlike physicians who continually update their knowledge and practice [38]. The above limitations have all been improved in GPT-4.0.

However, although GPT-4.0 has been upgraded and improved compared with GPT-3.5, things could still be improved. GPT-4.0 has limitations in identifying symptoms and laboratory test data by classifying and analyzing causes of misdiagnosis. First, like GPT-3.5, GPT-4.0 has limitations in training data scope and clinical context, and experience affects the model’s ability to recognize and interpret specific medical data accurately. It may need more specific medical data, impacting its ability to accurately identify and analyze complex medical symptoms and laboratory results. Second, GPT-4.0 lacks clinical context and cannot accurately understand the clinical context in which medical data is presented [21,39]. For instance, interpreting laboratory results requires understanding the numbers and the clinical context including patient history and physical examination findings. Third, GPT-4.0 is a general model, not adjusted explicitly for medical applications [40]. While it can process medical information, it cannot replace a human medical professional’s nuanced judgment and experiential learning [41].

In summary, although the evaluation results of GPT-4.0 in various classification diagnostic accuracies are better than GPT-3.5, it still has certain limitations, and its accuracy in medical diagnosis should be viewed with caution. It is a supportive tool [42], not a replacement for professional medical advice and judgment. It has yet to fully reach the diagnostic ability of clinical doctors. In the future, it is still necessary to continue training and improving GPT-4.0’s ability to recognize patient symptoms and laboratory test data.

### Limitations

This study has several limitations. First, we did not evaluate the models in real clinical settings. The dynamics of real-time clinical environments, complex and often unpredictable patient presentations, intraoperative findings, real imaging data, and pathological sections differ markedly from the controlled, historical data sets we used. Consequently, our findings do not fully encompass the challenges and variances in actual clinical practice. Second, we compared the diagnostic capabilities of GPT-3.5 and GPT-4.0 with predefined diagnoses in the literature rather than assessing their performance against the actual diagnostic abilities of human surgeons. Third, the study only included GPT-3.5 and GPT-4.0 models. With the rapid advancement of AI, numerous other models, such as Google’s Bard and Microsoft’s Copilot, have emerged. Future research should consider incorporating a broader range of AI models to evaluate their diagnostic capabilities comprehensively. Additionally, given that colorectal cancer is a prevalent disease in the field of surgery, this study merely serves as an initial exploration of ChatGPT’s application in surgery, using colorectal cancer as an example. We also did not assess GPT-3.5 and GPT-4.0’s ability to judge disease severity. In the future, we intend to include a broader range of surgical diseases to explore the potential of GPT-assisted clinical applications in surgery fully. The different prompts can yield varying answers. In this study, we crafted a simple and direct prompt and conducted multiple tests with both models, as subtle variations can influence the accuracy and relevance. Standardizing prompts is crucial to ensure the comparability and reliability of AI diagnostic performance assessments.

Moreover, the diagnostic basis relied solely on textual patient records from existing literature, which only demonstrates GPT-3.5 and GPT-4.0’s ability to extract and analyze textual information. This study needs to include and evaluate imaging and pathology interpretation, which are critical components of comprehensive clinical diagnosis. Another limitation is that the patient records were originally in Chinese and were translated into English before querying GPT-3.5 and GPT-4.0. Both machine and human translations may only partially capture the nuances and details of the original records, potentially affecting the accuracy and completeness of the information presented to the models. Finally, the study’s design as a cross-sectional comparative study provides a lower level of evidence in the hierarchy of evidence-based medicine compared to randomized controlled trials. Future research should involve randomized controlled trials for more robust and reliable assessments of GPT-assisted clinical applications.

### Conclusions

This study shows that ChatGPT has potential in medical diagnosis and may serve as a tool to assist doctors in clinical diagnosis and improve work efficiency. Generally, the diagnostic accuracy of GPT-4.0 is better than that of GPT-3.5, indicating that the upgrade of the ChatGPT version is affected. However, GPT-4.0 still has limitations regarding patient symptoms and laboratory data recognition, which needs to be further studied in the dynamic clinical practice environment in the future.

## Appendix

Multimedia Appendix 1 The case prompt provided to ChatGPT.

Multimedia Appendix 2 Cohen Kappa statistic for GPT-3.5 or GPT-4.0 agreement.

Multimedia Appendix 3 Cohen Kappa statistic for senior surgeons agreement.

## Abbreviations

AI: artificial intelligence
CDS: clinical decision support
CMAPH: Chinese Medical Association Publishing House
ICD-11: International Classification of Diseases, 11th Revision
LLM: large language model
PLA: People’s Liberation Army

## Data Availability

The data sets generated during and analyzed during this study are available from the corresponding author upon reasonable request.
