# Supplementary material for: The Diagnostic Ability of GPT-3.5 and GPT-4.0 in Surgery: Comparative Analysis
Source: J Med Internet Res. 2024 Sep 10;26:e54985. doi: 10.2196/54985 (PMC11422746; doi:10.2196/54985)
Supplement: Multimedia Appendix 1 [file jmir_v26i1e54985_app1.docx]

**Multimedia Appendix 1.** The case prompt provided to ChatGPT.

A case is illustrated as follows (the case from the CMAPH public database):

An old female patient was admitted to the hospital due to dull pain in the right lower abdomen, bloody stool for 2 months, and a right abdominal mass for more than 1 month. The operation was successful, but the bladder was filled before closing the abdomen. Examination showed that the balloon of the double-lumen urinary catheter placed before the operation was ruptured, and the catheter slipped out of the urethra by itself. After the operation, hemocoagulase 1000 U was given by intramuscular injection every day for 2 days. The catheter was removed on the third day after surgery, and the patient could urinate by herself with a 24-hour urine volume of about 1200 ml. On the morning of the fourth day after surgery, swelling of both lower limbs was found, especially in the left lower limb. The patient had no chest tightness, shortness of breath, cough, or other symptoms, and the biochemical examination on the same day showed that the electrolytes and plasma proteins were normal. Venous ultrasound examination of the lower extremities showed slow blood flow in the deep veins of the lower extremities, and spontaneous angiography was observed, but no deep venous thrombosis was found. On the fifth postoperative day, the patient urinated 1800 ml by herself, and the swelling of the lower limbs was slightly reduced. On the 7th day after the operation, the swelling of both lower limbs gradually aggravated again, involving the perineum. Re-examination of the lower extremity venous ultrasound showed that the blood flow was still slow, and the bladder was filled. The indwelling catheter was placed again to drain 2500 ml of urine, and then the catheter was continuously opened for drainage. The swelling of both lower limbs was significantly relieved on the same day. Reexamination of the lower extremity venous ultrasound showed normal blood flow and no thrombosis. On the 12th day after the operation, low molecular weight heparin calcium was stopped, and the catheter was removed. The patient could urinate by herself, and an ultrasound examination showed that the residual urine volume of the bladder was 20 ml. After 6 months of follow-up, no lower limb edema recurred. Please suggest the most likely primary and secondary diagnoses without explanation based on the above information (Figure S1).

**
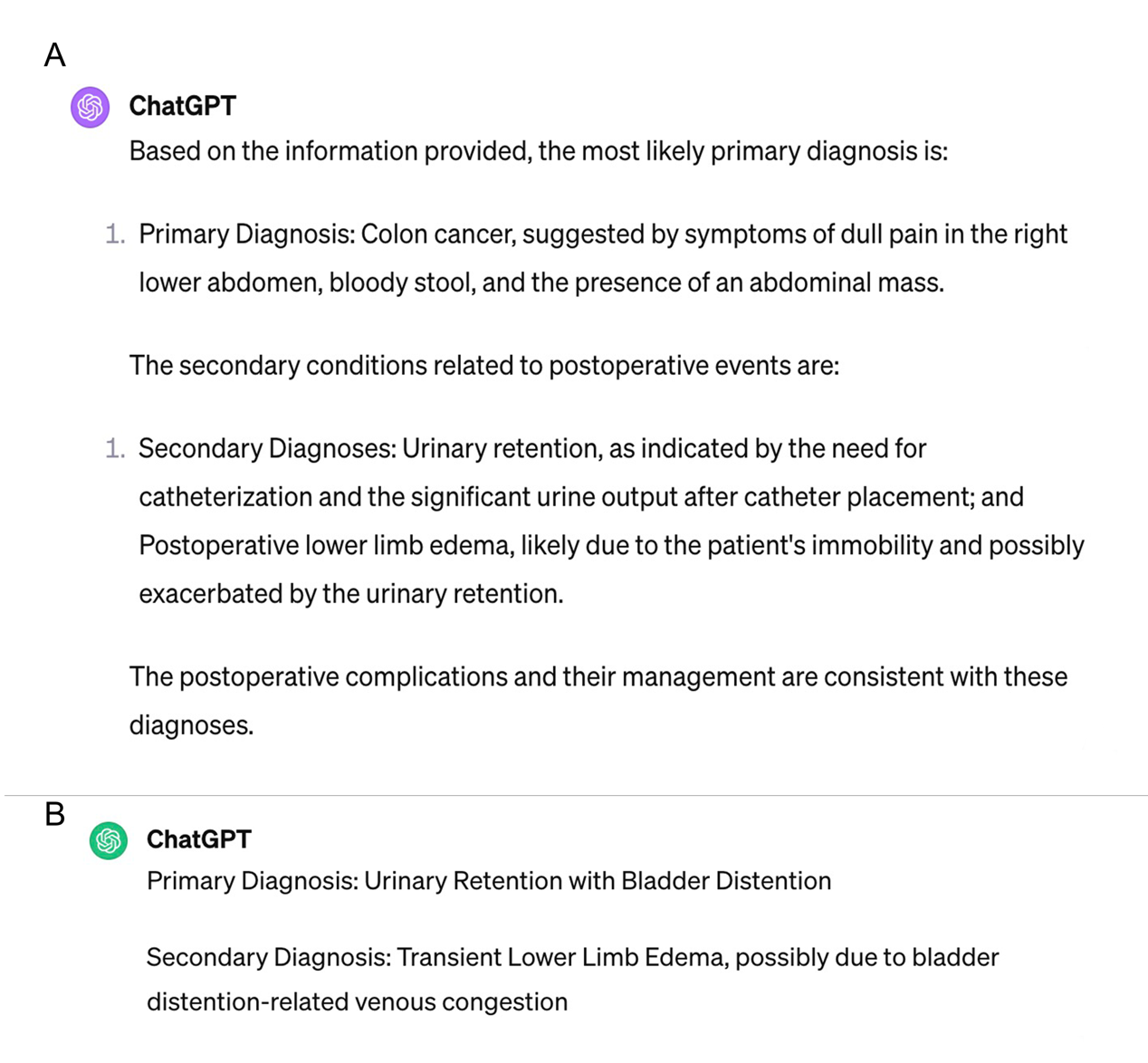
**

Figure S1 Screenshot of ChatGPT’s answer to a question (A) GPT-4.0; (B) GPT-3.5
