# Supplementary material for: The Diagnostic Ability of GPT-3.5 and GPT-4.0 in Surgery: Comparative Analysis
Source: J Med Internet Res. 2024 Sep 10;26:e54985. doi: 10.2196/54985 (PMC11422746; doi:10.2196/54985)
Supplement: Multimedia Appendix 2 [file jmir_v26i1e54985_app2.docx]

|  | **Cohen_κ value** | **% of agreement** | **N of cases** |
| --- | --- | --- | --- |
| **ChatGPT-4.0** |  |  |  |
| Primary Diagnosis | 0.528 | 95.8 | 286 |
| Secondary Diagnosis | 0.676 | 81.8 | 286 |
| **ChatGPT-3.5** |  |  |  |
| Primary Diagnosis | 0.859 | 94.4 | 286 |
| Secondary Diagnosis | 0.899 | 92.3 | 286 |

**Multimedia Appendix 2.** Cohen Kappa statistic for GPT-3.5 or GPT-4.0 agreement.
