# Supplementary material for: The Diagnostic Ability of GPT-3.5 and GPT-4.0 in Surgery: Comparative Analysis
Source: J Med Internet Res. 2024 Sep 10;26:e54985. doi: 10.2196/54985 (PMC11422746; doi:10.2196/54985)
Supplement: Multimedia Appendix 3 [file jmir_v26i1e54985_app3.docx]

|  | ChatGPT4.0 | | ChatGPT3.5 | |
| --- | --- | --- | --- | --- |
|  | Cohen κ value | p-value | Cohen κ value | p-value |
| Medical history | 1.000 | <0.0001 | 1.000 | <0.0001 |
| Symptoms | 0.972 | <0.0001 | 0.980 | <0.0001 |
| Physical examination | 0.980 | <0.0001 | 0.952 | <0.0001 |
| Laboratory tests | 0.984 | <0.0001 | 1.000 | <0.0001 |
| Imaging examination | 0.918 | <0.0001 | 0.986 | <0.0001 |
| Intraoperative findings | 0.971 | <0.0001 | 0.986 | <0.0001 |
| Pathology | 1.000 | <0.0001 | 1.000 | <0.0001 |

**Multimedia Appendix 3.** Cohen Kappa statistic for senior surgeons agreement.
